# Supplementary material for: Integrated analysis of the aqueous humor microbiome and lens capsule transcriptome in high myopia cataract: a pilot study
Source: Front Med (Lausanne). 2026 Jun 16;13:1845205. doi: 10.3389/fmed.2026.1845205 (PMC13314463; doi:10.3389/fmed.2026.1845205)
Supplement: Supplementary file 3 [file Data_Sheet_1.zip › supplemental_material/CN_method.pdf]

欧易生物 2bRAD-M

实验及分析流程（v202402）

oebiotech
欧易生物

2bRAD-M 实验在青岛欧易生物科技有限公司执行，具体步骤如下：

1 实验材料

1.1 试剂

BcgI(New England BioLabs, cat. no. R0545)

T4 DNA ligase (New England BioLabs, cat. no. M0202)

Phusion high-fidelity DNA polymerase (New England BioLabs, cat. no. M0530)

dNTP solution mix (New England BioLabs, cat. no. N0447)

DL2000 DNA ladder (Takara, cat. no. 3427)

100-bp DNA ladder (New England BioLabs, cat. no. N3231)

QIAquick PCR Purification Kit (Qiagen, cat. no. 28106)

Qubit dsDNA HS Assay Kit (Invitrogen, cat. no. Q3258)

Agarose (Sigma-Aldrich, cat. no. A0169)

10× TAE buffer (Sigma-Aldrich, cat. no. T8280)

5× TBE buffer (Sigma-Aldrich, cat. no. T3913)

CutSmart buffer (New England BioLabs, cat. no. B7204)

Oligonucleotides (Sangon Biotech)

Genomic DNA

Acrylamide/bis-acrylamide, 29:1 ratio (Sigma-Aldrich, cat. no. A2792)

Ammonium persulfate (APS; Sigma-Aldrich, cat. no. A3678)

N,*N*,*N*,*N*-Tetramethylethylenediamine (TEMED; Sigma-Aldrich, cat. no. T9281)

SYBR Safe DNA Gel Stain in DMSO solution (Thermo Fisher Scientific, cat. no. S33102)

1.2 仪器

MyCycler thermal cyclers (Bio-Rad)

Mupid-One agarose gel electrophoresis system (Takara)

Slab size PAGE system (ATTO)

ChemiDoc MP imaging system (Bio-Rad)

NanoVue Plus spectrophotometer (GE Healthcare)

Qubit 2.0 fluorometer (Invitrogen)

Centrifuge (Eppendorf, model no. 5427R)

ThermoMixer (Eppendorf, model no. F1.5)

1.5-ml Pellet pestle (Kimble)

2 实验方法

2.1 试剂准备

接头和引物 将合成的引物和接头干粉室温下高速离心，加水悬浮混匀制成浓度为 100 μM 的储存液。实验前制备浓度为 5 μM 的接头工作液和浓度为 10 μM 的引物，以上储存液和工作液于−20 °C 保存备用。

10% (wt/vol) APS 取 1 g APS 溶于少量水中，定容至 10 ml。该溶液可在-20°C保存 3-6 个月。

1% (wt/vol) 琼脂糖凝胶 称取 0.4 g 琼脂糖溶于 40 ml 1× TAE buffer，加热至琼脂糖完全融化，待溶液降温到约 60 °C 左右加入 4 μl SYBR Safe DNA 染料，摇匀后倒入事先插好梳子的胶槽中，室温放置，待胶凝固后使用。琼脂糖胶须现用现配。

8% (wt/vol) 聚丙烯酰胺凝胶 取 5.4 ml 丙烯酰胺 (29:1)、4 ml 5× TBE、280 μl 10% (wt/vol) APS、10 μl TEMED、10.6 ml 纯水轻摇混匀，然后倒入事先夹好的胶板中并插好梳子，室温下放置至少 1 小时后方可使用，8%聚丙烯酰胺凝胶须现用现配。

2.2 流程

酶切

下面要介绍的实验方法适用于单串联项目，实验操作时需谨慎，避免样品间交叉污染。

1 酶切反应体系如下，37 ℃ 反应 3h。

成分	体积(μl/单个样品；酶切组)
DNA (1pg-200ng)	x
10×cut smart buffer	1.5
BcgI （2U/μl）	2
Pure water	To 15

连接

2 连接反应体系如下，反应条件：4℃连接 12 h。

成分	体积(μl/单个样品)
酶切产物	10
10× T4 ligase buffer	1
Adaptor 1 (5μM)	0.8
Adaptor 2 (5μM)	0.8
T4 DNA ligase (400 U/μl)	2
Pure water	To 20

富集和纯化

3 PCR 反应体系如下

成分	体积(μl/单个样品)
连接产物	7
5×HF buffer	4
10 mM dNTP	0.6
Primer 1 (10 μM)	0.4
Primer 2 (10 μM)	0.4
Phusion high-fidelity DNA polymerase (2 U/μl)	0.2
Pure water	To 20

4 PCR 反应程序如下:

Cycle number	Denature	Anneal	Elongate

16-28	98℃,5s	60℃,20s	72℃,10s

5 将 20 µl PCR 产物和 1µl 100-bp DNA ladder 用 8%聚丙烯酰胺凝胶电泳检查，400 V 电泳 35 min。

6 电泳完毕后用 SYBR Safe DNA 染料染胶 3min，观察目的条带(100 bp)亮度。

7 切取目的条带放入 1.5ml 离心管，用研磨棒将胶磨碎后加入 30-40µl 纯水，于 4℃静置 6-12 h。

PCR 引入 Barcode 序列

8 PCR 反应体系如下：

成分	体积(µl)
连接产物	6
5×HF buffer	4
10 mM dNTP	0.6
5 µM 5UDI Primer	0.8
5 µM 7UDI Primer	0.8
Phusion high-fidelity DNA polymerase (2 U/µl)	0.2
Pure water	To 20

9 PCR 反应程序如下:

Cycle number	Denature	Anneal	Elongate
5-7	98℃,5s	60℃,20s	72℃,10s

10 用 QIAquick PCR 纯化试剂盒对 PCR 产物进行纯化，加 15µl 纯水洗脱后用 Qubit 定量。一般情况下，纯化产物的理想浓度为 10–30 ng/µl。

11 若已建好多个文库，可将不同 barcode 编号的文库按照送测数据量进行混库，最终混合好的文库浓度在 5–10 ng/µl 较适宜。

12 使用 Illumina Novaseq PE150 测序平台对混合好的文库进行测序。

附：

Table 1. Adaptors and primers used for 2bRAD-M library preparation.

Adaptors	Sequence (5’ to 3’)
Adap-1 sense	ACACTCTTTCCCTACACGACGCTCTTCCGATCTNN
Adap-1 antisense	AGATCGGAAGAGC
Adap-2 sense	GTGACTGGAGTTCAGACGTGTGCTCTTCCGATCTNN
Adap-2 antisense	AGATCGGAAGAGC
Primers	
Primer1	ACACTCTTTCCCTACACGACGCT
Primer2	GTGACTGGAGTTCAGACGTGTGCT
5UDI Primer	AATGATACGGCGACCACCGAGATCTACACXXXXXXXXACACTCTTTCCCTACACGACGCTCTTCCGATCT
7UDI Primer	CAAGCAGAAGACGGCATACGAGATXXXXXXXXGTGACTGGAGTTCAGACGTGTGCTCTTCCGATCT

3 分析流程

3.1 数据库构建

首先，获取 GTDB 及 Ensembl 数据库中的 404,199 个微生物基因组（包括细菌、真菌和古细菌），并对所有基因组进行电子酶切（16 种 2b 型限制性内切酶），获取特定分

类单元下的唯一标签（与该分类单元下其他物种没有重叠）作为物种特异性 2bRAD 标记，生成 2bRAD 微生物基因组数据库[1,2]。

3.2 相对丰度计算

将质控后的所有测序 2bRAD 标签映射到构建好的 2bRAD 标记数据库，使用下示公式计算每一个物种的 Gscore 值，筛选 Gscore 高于阈值 5 的物种作为候选物种，以此控制假阳性[2]。

$$\mathrm{G}\ score_{\ species\ i} = \sqrt{S_i \times t_i}$$

S：样本中映射到物种 i 的所有 2bRAD 标记的 Reads 数。

t：样本中映射到物种 i 的所有 2bRAD 标记的种类数。

然后，使用以下公式计算样本中每一个物种的相对丰度。

$$Relative\ abundance_{\ species\ i} = \frac{S_i/T_i}{\sum_{i=1}^{n} S_i/T_i}$$

S：样本中映射到物种 i 的所有 2bRAD 标记的 Reads 数。

T：数据库中物种 i 的所有 2bRAD 标记的种类数。

参考文献（请注意引用参考文献）

[1] Sun, Zheng, et al. "Removal of false positives in metagenomics-based taxonomy profiling via targeting Type IIB restriction sites." *Nature Communications* 14.1 (2023): 5321.

[2] Sun, Zheng, et al. "Species-resolved sequencing of low-biomass or degraded microbiomes using 2bRAD-M." *Genome biology* 23.1 (2022): 36.

青岛欧易生物科技有限公司
青岛市崂山区株洲路 143 号石老人科技创新园 3-B310
电话：0532-8870-6928

申明

本项目报告由青岛欧易生物科技有限公司提供给项目相关客户。本公司承诺：未经客户同意，不向第三方泄露数据及数据分析内容，不将客户数据用于任何商业行为（遵循合同保密协议）。客户未经本公司同意，不得以任何目的向第三方出示项目报告。本报告的最终解释权归青岛欧易生物科技有限公司。
